# Notes and News

**Published:** 1904-07-02

**Authors:** 


### NOTES AND NEWS.

The King, accompanied by the Queen, will visit St. Bartholomew's Hospital and lay the foundation stone for a new out-patient department on July 7th.

The Duchess of Albany will open the new out-patients' department in the ophthalmic department at the Royal Hospital, Richmond, on July 8th. She will receive purses for the Building Fund.

The Metropolitan Hospital Sunday Collection has nearly reached £40,000.

The late Mr. Frederick Midgeley, of Egerton, has bequeathed £1,000 to the Huddersfield Infirmary.

It is expected that about £8,000 will find its way to the Royal Victoria Hospital for Children, as the result of the bazaar and ball held at the Royal Albert Hall.

In response to several requests the portrait of Mr. George Herring, which was published with our Hospital Sunday Supplement of June 11th, has been reproduced on best art paper. Copies can be procured, price 6d., from The Scientific Press, 28 Southampton Street, Strand.

We publish in our "Letter Box" an account of the calamity which has befallen the Earlswood Asylum. It would be a national misfortune were the work of this institution to be curtailed, and so we hope that all who can possibly afford to do so will come forward with immediate financial assistance that the buildings may speedily be renovated and rendered secure for habitation.

Dr. and Mrs. Bailey and Mr. and Mrs. R. W. Baxter were "At Home" on June 29th to the vice-presidents and members of the Brentford District of the League of Mercy and others interested in its work, at Featherstone Hall and South Lodge, Southall. Dr. Bailey and Mr. Baxter are vice-presidents of the district, which has, since 1901, contributed £413 to the London hospitals through the League.

The British Homœopathic Association will hold a fête in the Botanic Gardens on July 7th.

The annual banquet of the Poor Law Medical Officers was held on Tuesday at the Trocadero Restaurant.

Lord Alverstone distributed the prizes to the students of the Medical School at St. Mary's Hospital on Wednesday.

On July 5th an entertainment will be given at Her Majesty's Theatre in aid of the Ophthalmic Hospital of the Order of St. John of Jerusalem.

A concert will be held on July 7th at St. James's Hall, in aid of the Italian Hospital. The concert is organised by the Italian Chamber of Commerce.

The Home Office has consented to allow the Joyce Green Hospital of the Metropolitan Asylums Board to be registered as a place where experiments on living animals may be performed. The consent is accompanied by conditions as to those who will be able to exercise the powers.

Professor Clifford Allbutt expressed the feelings of many reasonable people when addressing the recipients of prizes at the London School of Medicine for Women. He said the time had come when, in his opinion, women should be admitted as members of the Colleges of Physicians and Surgeons.

At the annual dinner of the Post-Graduate College of the West London Hospital it was announced that amongst the entries of the past year were numbered the names of naval men on leave, members of the Army Medical Corps, the Indian Medical Service, and of a good many medical practitioners in and around London.

A Hospital for Consumption has been opened by the Lady Mayoress for Leeds. Armley House, a private residence, has been secured for the purpose, and initially accommodation will be provided for 20 patients. The hospital will work in connection with the Leeds Association for the Care and Prevention of Tuberculosis. Mr. Henry Barran has generously undertaken to pay the rent of the house for three years.

The work which the Duchess of Albany helped to forward when she opened a sale of work by the inmates of the Paddington Workhouse is a very excellent one. The Brabazon Employment Society, established by Lady Meath when Lady Brabazon, serves to give interesting employment to the aged and infirm in our workhouses. The institution is self-supporting, as the work executed by both the men and women is sold for the purpose of procuring fresh materials. Thus the society introduces an interest into sadly-monotonous lives, and enables the workers to feel that they have still the power of helping others. There are already about 1,800 voluntary teachers employed in helping the old people to take up some form of light and interesting manual labour. Three hundred workhouses are connected with the scheme, and it is to be hoped that all will eventually benefit before long.

